# CRISPR/Cas9-mediated knockout of *DFR* alters pigmentation and shifts flavonoid accumulation in red leaf lettuce without detectable growth penalties

**DOI:** 10.3389/fgeed.2026.1755922

**Published:** 2026-03-04

**Authors:** Ai Nagamine, Masaki Ono, Osuke Sato, Eiji Goto, Hiroshi Ezura

**Affiliations:** 1 Institute of Life and Environmental Sciences, University of Tsukuba, Tsukuba, Japan; 2 Graduate School of Horticulture, Chiba University, Matsudo, Japan; 3 Graduate School of Life and Environmental Sciences, University of Tsukuba, Tsukuba, Japan; 4 Tsukuba Plant Innovation Research Center, University of Tsukuba, Tsukuba, Japan

**Keywords:** anthocyanin, CRISPR-Cas9, DFR (dihydroflavonol 4-reductase), flavonol, genome editing, lettuce, plant factories with artificial light (PFALs)

## Abstract

Red leaf lettuce (*Lactuca sativa* L. cv. ‘Red Fire’) is a preferred crop in plant factories with artificial light (PFALs) due to its short cultivation cycle and high anthocyanin content, which increases both its nutritional value and visual appeal. However, anthocyanins strongly influence leaf coloration and antioxidant profiles, and their levels are highly responsive to the light environment. Therefore, targeted editing of flavonoid biosynthesis may provide a breeding strategy to diversify pigment composition and associated functional traits under PFAL conditions. In this study, we used CRISPR/Cas9 to knock out *DFR* (dihydroflavonol 4-reductase), a key enzyme in the anthocyanin pathway. Genome-edited lines were generated via a dual-guide RNA system, resulting in a successfully edited red leaf genotype. The *DFR*-knockout lines displayed a complete loss of red pigmentation and a visibly distinct green phenotype. Metabolite profiling revealed a significant decrease in anthocyanin levels, accompanied by an increase in total flavonoid levels in some lines. Growth traits, including shoot dry weight and leaf number, were not significantly affected, suggesting that *DFR* knockout does not compromise growth under PFAL conditions. These findings highlight *DFR* as a promising target for creating pigment-altered lettuce lines for controlled-environment cultivation, including PFAL systems.

## 1 Introduction

Plant factories with artificial light (PFALs) have been gaining attention as a potential solution for addressing climate change issues in agriculture. Although PFALs offer advantages such as the ability to cultivate crops independently of external environmental conditions, they also face challenges in terms of initial costs (e.g., facility investment) and running costs (e.g., electricity bills). Currently, PFALs primarily produce leafy vegetables such as lettuce, which are suitable for this system due to their short growth cycle, compact form, and light weight (Kozai, 2013). However, because the high production cost of PFAL crops is not easily reflected in their retail prices, they remain less economically competitive than field-grown vegetables are (Kozai and Niu, 2019).

One promising approach to overcome this economic issue is the development of high-value-added crops that are either difficult to produce in open fields or can justify a higher price. Leaf lettuce is among the main crops grown in PFALs due to its short growth cycle, low height, and good compatibility with market demands in Japan (Ministry of Agriculture, Forestry and Fisheries, “Survey on Large-scale Greenhouse Horticulture and Plant Factories,” 2021). It is generally consumed raw, such as in salads. For these reasons, leaf lettuce is also considered a useful model crop for breeding tailored to PFALs. In particular, improving the nutritional content and composition of edible parts is a major goal in the development of new cultivars.

**FIGURE 1:**
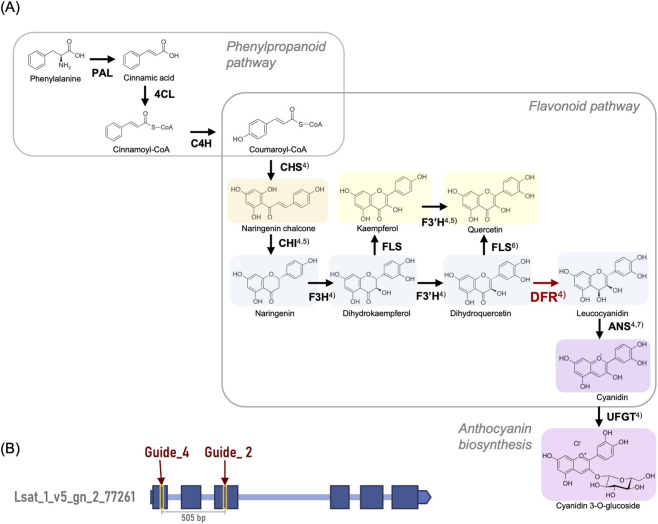
The target gene for modification of flavonoid metabolism by CRISPR/Cas9 knockout. **(A)** Proposed biosynthetic pathway of flavonoids and anthocyanins in red leaf lettuce. This model is primarily based on previous studies in *Arabidopsis thaliana* and Lactuca sativa. Arrows indicate the direction of enzymatic catalysis, with each arrow positioned next to the corresponding enzyme. The color highlights of each flavonoid compound reflect their approximate natural pigment colors. The key enzyme analyzed in this study, DFR (Dihydroflavonol 4-reductase), and its associated reaction are shown in red. Abbreviations of the other enzymes used in the figure are as follows: PAL (Phenylalanine Ammonia-Lyase), 4CL (4-Coumarate:CoA Ligase), C4H (Cinnamate-4-Hydroxylase/CYP73A), CHS (Chalcone Synthase), CHI (Chalcone Isomerase), F3H (Flavanone 3-Hydroxylase), FLS (Flavonol Synthase), ANS (Anthocyanidin Synthase) and UFGT (UDP-glucose: flavonoid 3-O-glucosyltransferase). Superscript numbers indicate references used to construct the pathway model: Liu et al., 2021; Winkel-Shirley, 2001; Saito et al., 2013; Zhang et al., 2018; Gurdon et al., 2019; Ebisawa et al., 2008; Gurdon et al., 2021. **(B)** Gene structure of the *DFR* gene and CRISPR–Cas9 guide RNA target sites. The gene corresponds to Lsat_1_v5_gn_2_77261 (2,340 bp) in the lettuce genome version 8 annotation. Each dark blue box shows an exon or untranslated region (UTR), and each light blue line shows an intron. Two guide RNA target sites (Guide_4 and Guide_2) are highlighted with yellow boxes and indicated by dark red arrows. The distance between Guide_4 and Guide_2 is 505 bp.

Among the target compounds, anthocyanins—responsible for red pigmentation in red leaf lettuce—are secondary metabolites derived from the flavonoid biosynthesis pathway that starts from p-coumaroyl CoA (Winkel-Shirley, 2001; Ebisawa et al., 2008; Gurdon et al., 2021; Gurdon et al., 2019; Liu et al., 2021; Saito et al., 2013; Zhang et al., 2018) (Figure 1A). These compounds are valued for their health-promoting antioxidant activity. However, anthocyanins absorb part of the visible light spectrum and contribute to photoprotection, potentially influencing light distribution within leaves depending on the growth environment (Zhang et al., 2018).

Dihydroflavonol 4-reductase (DFR), a key enzyme involved in this pathway, catalyzes the conversion of dihydroflavonols (such as dihydrokaempferol and dihydromyricetin) into anthocyanidin precursors. DFR shares these substrates with flavonol synthase (FLS), which converts them into flavonols. These two enzymes compete for the same substrates (Zhang et al., 2016; Figure 1A). Therefore, knocking out DFR is expected to suppress anthocyanin biosynthesis while promoting the accumulation of flavonols by redirecting the metabolic flux. This may lead to a dual effect: (1) altering pigment composition and visual traits and (2) increasing flavonol intermediates with enhanced nutritional value.

In addition, modulating anthocyanin biosynthesis provides an opportunity to diversify pigment composition and flavonoid profiles under controlled lighting conditions. For this reason, *DFR* knockout could become a promising breeding target, especially for PFAL-oriented leaf lettuce, where light quality, light intensity, and other environmental factors can be strictly controlled.

Although loss-of-function mutations in genes such as *CHI* (*chalcone isomerase*), *F3′H* (*flavanone 3′-hydroxylase*), and *ANS* (*anthocyanidin synthase*) have been shown to increase the accumulation of specific flavonoids such as naringenin chalcone, kaempferol, and quercetin (Gurdon et al., 2021; Gurdon et al., 2019; Zhang et al., 2018), the specific role of DFR in lettuce and its broader impact on flavonoid metabolism remain unclear.

Traditional breeding through crossing requires significant time and effort to introduce desirable traits. In contrast, genome editing is a powerful tool that allows the introduction of target traits into existing cultivars in a relatively short time while avoiding the loss of beneficial traits or the introduction of unwanted ones (Nagamine and Ezura, 2024). Among genome editing methods, SDN-1 (site-directed nuclease-1), which causes small insertions or deletions at the target site, is especially notable. 1n Japan, SDN-1-edited crops are not considered genetically modified organisms (GMOs) under the domestic implementation of the Cartagena Protocol. However, regulations currently vary by country: such crops are treated as GMOs in the European Union but are often exempt from regulation in the United States and Argentina (Ezura, 2022; Kawall and Kozai, 2021; Nagamine et al., 2023; Nagamine and Ezura, 2022).

The first commercially available genome-edited crop using CRISPR-Cas9 was the high-GABA tomato ‘Sicilian Rouge High GABA’ developed in Japan (Sanatech Seed), followed by a waxy corn line with *Wx1* deletion announced in 2023 (USDA Foreign Agricultural Service, 2023; ISAAA, 2023). Other examples include CRISPR/Cas9-edited tomato with *SGR1* knockout to increase lycopene biosynthesis via chloroplast retention (Li et al., 2018) and lettuce lines with *LCY-ε*, *uGGP1*/*uGGP2*, and *CCD4a* edited to increase beta-carotene, zeaxanthin, and ascorbic acid contents (Livneh et al., 2025). These cases highlight the effectiveness of genome editing in efficiently introducing target traits.

In this study, we aimed to evaluate the effects of knocking out the *DFR* gene using CRISPR/Cas9, focusing on changes in pigment composition, flavonoid metabolism, and growth characteristics in red leaf lettuce. In particular, we examined whether disruption of anthocyanin biosynthesis could redirect flavonoid metabolic flux toward other flavonoid pools in edible tissues. A green leaf cultivar (‘Green Wave’), which intrinsically lacks anthocyanin accumulation, was included as a reference line to evaluate genome-editing efficiency and to confirm that *DFR* knockout itself does not cause unintended phenotypic effects independent of anthocyanin biosynthesis.

## 2 Materials and methods

### 2.1 Plant material

Red leaf lettuce (*Lactuca sativa* L. cv. ‘Red Fire’, Takii Seed Co., Kyoto, Japan) and green leaf lettuce (*L. sativa* cv. ‘Green Wave’, Takii Seed Co., Kyoto, Japan) were used in this study.

### 2.2 Guide RNA selection

Two guide RNAs (Guide_2 and Guide_4) for the genome editing of the *DFR* gene (Lsat_1_v5_gn_2_77261) were selected from the candidates predicted by CRISPR-P v2.0 (http://crispr.hzau.edu.cn/CRISPR2/), with an On-Score of 0.5 or higher, a low predicted off-target, a G/C content of 30%–80%, and a guide RNA secondary structure score within the acceptable range. The reference *DFR* sequence used for guide RNA design was obtained from the *Lactuca sativa* reference genome (Phytozome v8; https://phytozome-next.jgi.doe.gov/info/Lsativa_V8), which is based on cv. Salinas, a green-leaf lettuce cultivar. Despite the lack of anthocyanin accumulation in this cultivar, the *DFR* coding sequence retains all conserved motifs required for enzymatic activity. The position of each guide RNA on the gene is shown in Figure 1B. The sequence of each guide RNA used is shown in Supplementary Table S1.

The *DFR* gene (Lsat_1_v5_gn_2_77261) was identified as the sole *DFR* ortholog in the lettuce genome based on BLASTP searches using previously characterized DFR protein sequences from *Arabidopsis thaliana*, *Solanum lycopersicum*, and *Vitis vinifera* as queries against the *Lactuca sativa* genome database (Phytozome v5 and v8). Only a single locus showed high sequence similarity and conserved catalytic motifs characteristic of DFR enzymes.

### 2.3 Vector construction and tomato transformation

The vector pDeCas9-Kan (Fauser et al., 2014; Mikami et al., 2015) was used for genome editing of the *DFR* gene by CRISPR-Cas9. These plasmids were modified in accordance with methods presented in previous studies (Fauser et al., 2014; Mikami et al., 2015). The constructed vectors were subsequently introduced into *Agrobacterium tumefaciens* GV2260 *via* electroporation. Lettuce transformation was carried out with the Lettuce-optimized protocol (Pileggi et al., 2001), and then the regenerated diploid shoots that were rooted under selection medium (1/2 M medium containing 1.5% (w/v) sucrose, 50 μg/mL kanamycin, 375 μg/mL Augmentin, and 0.3% (w/v) Gelrite, pH 5.6–5.8) were selected as T_0_ plants.

### 2.4 Plant cultivation

The selected T_0_ plants were cultivated in a growth room at the University of Tsukuba, Japan, under controlled conditions: 25 °C, fluorescent lighting at 300 μmol m^-2^·s^-1^, and a 16 h light/8 h dark photoperiod. The plants were subsequently grown until line-specific indels were confirmed, after which the T_1_ seeds were harvested. During this period, a standard nutrient solution (Otsuka A, Otsuka Chemical Co., Ltd., Osaka, Japan) was supplied (approx. final EC = 1.4 dS m^-1^; pH = 6.5).

T_1_ seeds were germinated and transferred onto rockwool blocks (5 × 5 × 5 cm) and further cultivated under the same conditions after the indel genotypes were confirmed. T_2_ plants used for brief phenotyping (Figure 3) were also grown under the same conditions.

For the growth assessment and metabolite analysis, T_2_ plants were grown in a closed plant production system with multilayer cultivation shelves at Chiba University, Japan. Seeds were sown on filter paper moistened with tap water and transferred to urethane sponges (3 × 3 × 3 cm) at 2 days after sowing (DAS). At 9 DAS, the seedlings were placed on floating cultivation panels (M Hydroponic Research Co., Ltd., Aichi, Japan) in cultivation containers (TC-107, 39.8 × 27.7 × 12.4 cm, Sekisui Techno Molding Co., Ltd., Tokyo, Japan) filled with 1.8 L of nutrient solution and subsequently cultivated until 23 DAS.

A modified OAT hydroponic nutrient solution (OAT Agrio Co., Ltd., Tokyo, Japan) was used. Stock solutions were prepared in deionized water at the following concentrations: OAT Solution 1 (150 g/L), OAT Solution 2 (100 g/L), and OAT Solution 5 (25 g/L). A quarter-strength working solution was prepared by mixing 150 mL of Solution 1, 150 mL of Solution 2, and 60 mL of Solution five into 60 L of tap water (approx. final EC = 0.85 dS m^-1^; pH = 6.5).

The cultivation conditions in the PFAL included a photosynthetic photon flux density (PPFD) of 200 μmol m^-2^·s^-1^ at the panel surface, a CO_2_ concentration of 1,000 μmol mol^-1^, a day/night temperature of 25/20 °C, a relative humidity of 70%, and a 16 h light/8 h dark photoperiod. Neutral-white LED lamps (LT-B4600T08-N, 5000 K, OHM Electric Inc., Saitama, Japan) were used as the light source.

### 2.5 Indel detection

Indel detection was performed in the T_0_ generation using all regenerated, rooted plants that were confirmed to carry T-DNA insertions. Genomic DNA was extracted from young leaf tissues of each transformant. The presence of T-DNA was first confirmed by PCR amplification using *Cas9*-specific primers (Cas9 F1/R2; Supplementary Table S1). For indel detection at the target sites, PCR fragments were amplified using target site–specific primers (Supplementary Table S1) and subsequently sequenced by the Sanger method using the same primers. For T_1_ and subsequent generation plants, genomic DNA was similarly extracted from young leaf tissues to confirm both the absence of residual T-DNA and the inheritance of indel mutations at the target sites.

### 2.6 Growth assessment of T_2_ lines

Destructive analysis of T_2_ plants was conducted at 21 DAS. All leaves of each T_2_ plant were photographed to record their external morphology, and the shoot was subsequently harvested and dried at 70 °C for 72 h to determine its dry weight. Two or three biological replicates were used per line. With respect to shoot dry weight, because the sample size was limited (n = 2–3 per line), we present descriptive statistics together with 95% bootstrap confidence intervals (10,000 resamples), without formal hypothesis testing. Pairwise Welch’s t-tests were performed only as exploratory comparisons, and the resulting “NS” labels in the figure indicate that no clear differences were detectable at this sample size.

### 2.7 Determination of total phenolics, flavonoids and anthocyanins

To analyze the bioactive compounds, the fifth leaves of the lettuce plants were sampled. Leaves were counted starting from the bottom, excluding cotyledons. The collected leaf samples were stored in a deep freezer (−80 °C) until further analysis. The total phenolic concentration was analyzed using a slightly modified method by Ainsworth and Gillespie (2007). The flavonoid concentration was determined using the method of Zhishen et al. (1999). The concentration of anthocyanins in red lettuce was measured with slight modifications to the analytical method of (Mancinelli and Schwartz (1984). Fresh leaf samples (approximately 50 mg each) were used for the analyses. The remaining analytical procedures for determining the total phenolic, total flavonoid and anthocyanin contents are described in Lee and Goto (2022).

The total phenolic concentration was expressed as milligrams of gallic acid equivalents (GAE) per gram of dry weight (GAE mg g^–1^ DW). The flavonoid concentration was expressed as milligrams catechin equivalents per gram dry weight (mg catechin/g DW). The concentration of anthocyanins is presented as micrograms of cyanidin-3-glucoside per gram of dry weight (μg C3G g^−1^ DW). Statistical analysis was performed using one-way ANOVA followed by the Tukey–Kramer multiple comparison test (p < 0.05). Different letters indicate significant differences among groups.

## 3 Results

### 3.1 CRISPR-Cas9 genome editing efficiency of the *DFR* gene in red leaf lettuce ‘Red Fire’

In this study, we performed CRISPR-Cas9 genome editing to generate loss-of-function mutations in the *DFR* gene. The construct used was a CRISPR-Cas9 binary vector routinely employed in the Laboratory of Vegetable and Floriculture Science at the University of Tsukuba. Transformation was carried out via *Agrobacterium tumefaciens*, targeting the cotyledons of red leaf lettuce ‘Red Fire’ and green leaf lettuce ‘Green Wave’ (a vector map is shown in Supplementary Figure S1).

The target gene, *DFR,* was confirmed to be a single-copy gene through a homology search using the NCBI and Phytozome databases. Two gRNA target sites were selected using CRISPR-P v2, both located near the 5′ end of the coding sequence and spaced approximately 505 bp apart. This design allowed for not only individual indel formation but also the possibility of large deletions between the two sites.

After transformation, regenerated shoots were induced via callus formation and selected after rooting. Genomic DNA was extracted to confirm T-DNA integration and indel formation. The numbers of explants and regenerated lines at each stage are summarized in Table 1.

**TABLE 1:** Transformation and regeneration efficiency in this experiment.

Lettuce cultivar	Seeds	Inoculated cotyledon	Callus	Shoots	Rooted	T-DNA insertion	Indel detected
Green wave (control)	100	373	162	86	50	26	6
Red fire	100	381	265	38	12	5	1

Numbers represent the number of explants or individuals at each stage, and the number of plants with confirmed T-DNA, insertion or indels.

Compared with ‘Green Wave’, ‘Red Fire’ showed lower values for shoot regeneration, number of rooted lines, T-DNA-positive lines, and indel detection. However, owing to the limited number of regenerated plants, statistical significance could not be evaluated, and it is currently difficult to conclude that there is a clear varietal difference. In total, six independent *T*_*0*_ lines with confirmed indels were obtained for ‘Green Wave’, whereas only one such line was obtained for ‘Red Fire’. These results suggest a tendency toward lower transformation and genome editing efficiency in red leaf lettuce varieties.

### 3.2 Mutations introduced into the *DFR* gene and their structural impact

In this study, the lettuce dihydroflavonol 4-reductase (*DFR*) gene was targeted using a CRISPR-Cas9 system with two guide RNAs, Guide_2 and Guide_4, for genome editing (Figure 1B). Among eight T_0_ lines (one from ‘Red Fire’ and seven from ‘Green Wave’), several individuals exhibited either a +1 bp insertion or a −9 bp deletion, primarily at the respective Cas9 cleavage sites (Table 2). These mutations were stably inherited in the T_1_ and T_2_ generations (Table 3). Notably, T_2_ lines such as R1-11, R1-25, and R1-62 exhibited homozygous mutations at both target loci.

**TABLE 2:** Indels of T_0_ lines.

Line no.	Guide4		Guide2		Original cultiver
	Indel or subst	Genotype	Indel or subst	Genotype	
R1	+1/-9	Biallelic	+1/0	Hetero	Red fire
G13	+1/T→G	Biallelic	+1/0	Hetero	Green wave
G14	+1/T→G	Biallelic	+1/0	Hetero	Green wave
G27	+1	Homo	+1/0	Hetero	Green wave
G29	+1	Homo	+1/0	Hetero	Green wave
G30	+1	Homo	+1/0	Hetero	Green wave
G38	+1	Homo	+1/0	Hetero	Green wave

subst: substitution, Hetero: Heterozygous, Homo: Homozygous.

**TABLE 3:** Indels of T_1_ and T_2_ lines.

Line no.	Guide4		Guide2		Generation	Original cultiver
	Indel or subst	Genotype	Indel or subst	Genotype		
R1-11	+1	Homo	+1	Homo	T2	Red fire
R1-25	+1	Homo	+1	Homo	T2	Red fire
R1-62	−9	Homo	+1	Homo	T2	Red fire
R1-65	−9	Homo	+1	Homo	T2	Red fire
R1-72	+1	Homo	+1	Homo	T2	Red fire
R1-75	−9	Homo	+1	Homo	T2	Red fire
R1-78	−9	Homo	+1	Homo	T2	Red fire
R1-83	+1	Homo	+1	Homo	T2	Red fire
G27	+1	Homo	+1/0	Hetero	T1	Green wave
G29	+1	Homo	+1/0	Hetero	T1	Green wave
G30	+1	Homo	+1/0	Hetero	T1	Green wave

Despite the simultaneous use of dual guide RNAs, no large deletions spanning either the Guide_2 or Guide_4 site (approximately 505 bp apart) were detected in any of the lines analyzed.

**FIGURE 2:**
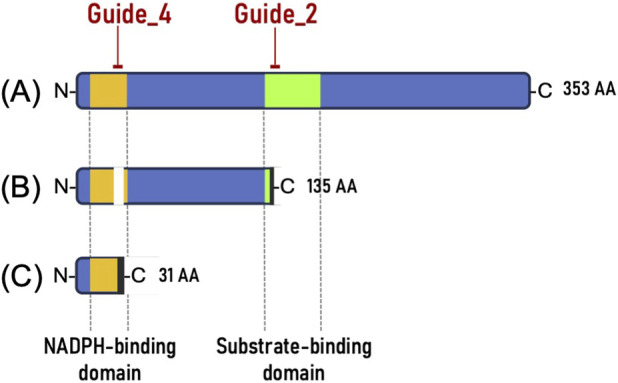
Schematic of the mutation pattern of the DFR protein. The conserved functional domains—the NADPH-binding domain and the substrate-binding domain—are highlighted in yellow and green boxes, respectively. The domain boundaries were verified using InterProScan and defined with reference to the conserved motifs and structural features reported in Choudhary and Pucker (2024), Li et al. (2023), Ruan et al. (2022) and Zhang et al. (2025). **(A)** Wild-type DFR protein. **(B)** Mutant type 1 with **(A)** 9 bp deletion at the Guide_4 site and a +1 bp insertion at the Guide_2 site. The white space indicates the deletion of three amino acids. **(C)** Mutant type 2 with a +1 bp insertion at the Guide_4 site.

To assess the functional consequences of the mutations, we predicted structural alterations in the DFR protein based on amino acid translations and domain annotations (Figure 2). The −9 bp deletion at Guide_4 led to the loss of three amino acids within the NADPH-binding domain, potentially compromising cofactor binding. In contrast, the +1 bp insertion at Guide_2 induced a frameshift mutation, resulting in a disrupted substrate-binding domain and premature translational termination. These structural changes suggest that the Guide_2 mutation is highly likely to cause a complete loss of DFR enzymatic activity, whereas the Guide_4 mutation may impair enzymatic function by destabilizing cofactor interactions or protein folding.

### 3.3 Phenotype of the *DFR-*KO ‘Red Fire’ T_2_ lines

**FIGURE 3:**
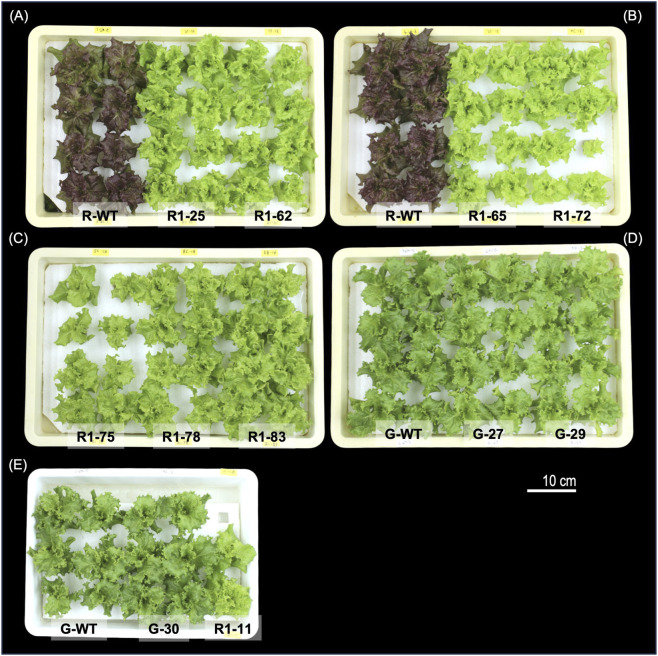
Plant phenotypes of *DFR*-knockout lines (T_2_ and T_1_ generations). Top-view photographs of individual plants from different lines at 35 DAS are shown in panels **(A–E)**. In all trays except for **(E)**, eight plants (four per row × two rows) were cultivated per line. The lines derived from the red-leaf background are from the T2 generation, while those from the green-leaf background are T1 plants. In panel **(E)**, three plants per row were grown for the G-WT and G-72 lines. Due to limited seed availability, only two plants (one row) were cultivated for line R1-11.

As shown in Figure 2, the leaf color of the genome-edited R1 line derived from ‘Red Fire’ clearly differed from that of the wild type (R-WT). The reddish-purple color caused by the anthocyanins had disappeared, and the leaves had turned fully green (Figures 3A–C, E). This visual change suggests a significant reduction in anthocyanin accumulation, likely due to the loss of *DFR* gene function.

Because R1 was the only genome-edited line obtained in the T_0_ generation, we grew its T_1_ progeny and selected lines including T-DNA-positive plants. The results revealed either a 1-bp insertion or 9-bp deletion at the Guide_4 target site and a 1-bp insertion at the Guide_2 site. All mutations were homozygous or biallelic. To evaluate stable phenotypes in a fixed genetic background, we selected T_2_ lines that were both T-DNA-free and genetically fixed (null segregants).

For comparison, we also cultivated the wild-type ‘Red Fire’ (R-WT: Figures 3A,B), the wild-type green leaf lettuce ‘Green Wave’ (G-WT), and its *DFR*-KO T_1_ lines (G-27, G-29, G-30) under the same conditions. The phenotypes and leaf colors were visually compared (Figures 3D,E).

All the T_2_ lines derived from ‘Red Fire’ (R1-25, R1-61, R1-65, R1-72, R1-75, R1-78, R1-83, and R1-11) completely lost the reddish-purple color seen in the wild type and turned green (Figures 3A,B,E). In contrast, no obvious color differences were detected between the wild-type and *DFR*-KO lines in ‘Green Wave’ (Figures 3D,E). This may be because ‘Green Wave’ originally does not accumulate anthocyanins, and *DFR* knockout did not have additional effects on leaf color.

In the *DFR*-KO lines of ‘Green Wave’, the plant morphology did not noticeably differ from that of the wild type (Figures 3D,E). However, in the genome-edited T_2_ lines of ‘Red Fire’, some plants tended to have a slightly more compact form than R-WT did (Figures 3A,B,E).

**FIGURE 4:**
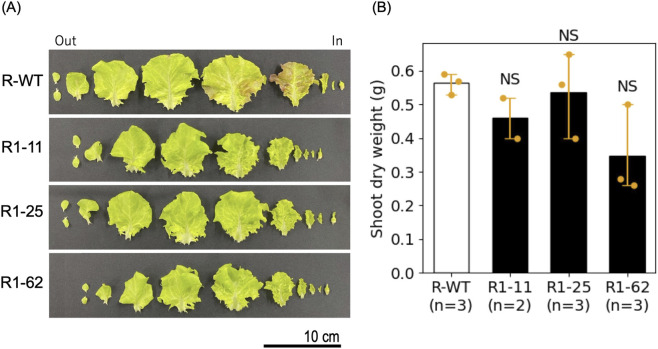
Growth assessment of T_2_ lines at 21 days after sowing (DAS). **(A)** Representative leaf morphology of each T_2_ line. Leaves are arranged from the outermost (first leaf, left) to the innermost (10th or 11th leaf, right) for each line. R-WT: wild-type; R1-11, R1-25, R1-62: genome-edited lines. **(B)** Shoot dry weight of each T_2_ line at 21 DAT. Data represent individual biological replicates (n = 2 for R1-11 and 3 for R1-25 and −62). Bars indicate the mean shoot dry weight; error bars represent 95% bootstrap confidence intervals (10,000 resamples). Dots (orange) represent individual plants. Pairwise comparisons using Welch’s t-test were performed as exploratory analyses only, and results should be interpreted with caution.

To quantify this tendency, we conducted a destructive growth analysis at the cultivation stage 21 days after sowing. We measured the number of true leaves, the leaf development pattern, and the above-ground dry weight as a yield index (Figure 4A). As a result, R1-11 and R1-25 showed dry weights comparable to the wild type, suggesting no apparent differences in growth performance under the present conditions (Figure 4B). In contrast, R1-62 showed a slight reduction in dry weight, although the magnitude of reduction was small (Figure 4B). These observations suggest that the effects of *DFR* loss-of-function on growth traits or yield may vary among lines under the present conditions.

### 3.4 Quantification of total phenolics, flavonoids, and anthocyanins in T_2_ lines

**FIGURE 5:**
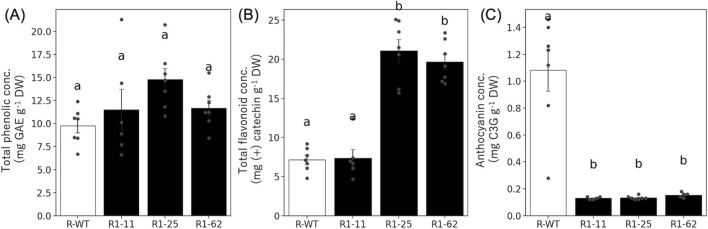
Quantification of total phenolics, total flavonoids and anthocyanin in T_2_ Lines. Mean concentrations of **(A)** total phenolics, **(B)** total flavonoids, and **(C)** anthocyanins in the fifth leaf of lettuce T_2_ lines at 20–23 DAS: DAT (n = 5–7). Each error bar shows SE. Different letters indicate significant differences among groups (one-way ANOVA followed by Tukey–Kramer test, p < 0.05).

To evaluate the effect of *DFR* gene knockout on flavonoid metabolism in lettuce, we collected the 5th leaves at 20–23 DAS and quantified the total phenolics, total flavonoids, and anthocyanins (n = 5–7; Figure 5).

The concentrations of total phenolics were slightly greater in all the genome-edited T_2_ lines (R1-11, R1-25, and R1-62) than in the wild-type ‘Red Fire’ (R-WT), but the difference was not significant (Figure 5A). In contrast, the total flavonoid concentrations were clearly increased in R1-25 and R1-62, suggesting a shift in metabolic flux in the flavonoid biosynthetic pathway, possibly due to the accumulation of upstream metabolites caused by the blockage of anthocyanin synthesis. In R1-11, the value was similar to that of the wild type (Figure 5B).

The concentration of anthocyanins was strongly decreased in all the genome-edited T_2_ lines to less than one-tenth of the wild-type level (Figure 5C). These results are consistent with the role of *DFR* as a key enzyme in anthocyanin biosynthesis.

In summary, *DFR* knockout effectively inhibited anthocyanin accumulation and, in some lines, influenced the accumulation of upstream flavonoid intermediates, likely including flavonols.

## 4 Discussion

### 4.1 Functional loss of DFR and visible phenotype confirmation

In this study, we used CRISPR/Cas9-mediated genome editing to knock out the *DFR* gene in red leaf lettuce (‘Red Fire’). This resulted in a complete loss of anthocyanin accumulation and a visible shift in leaf color from red–purple to green. These changes were consistently observed in multiple T_2_ lines, reaffirming the role of *DFR* as a rate-limiting enzyme in anthocyanin biosynthesis at the whole-plant level.

Notably, the introduced mutations were located within the predicted NADPH-binding and substrate-binding domains based on sequence homology (Supplementary Figure S2). Even relatively small changes, such as a +1 bp insertion or −9 bp deletion, led to complete loss of function. These findings provide the first experimental evidence supporting the functional significance of these domains in *Lactuca sativa*.

The reference *DFR* sequence used for guide RNA design was derived from *Lactuca sativa* cv. Salinas, a green-leaf lettuce cultivar that does not accumulate anthocyanins. Despite this, the DFR protein retains all conserved motifs required for enzymatic activity, indicating that the absence of anthocyanin pigmentation in green lettuce is not due to defects in the *DFR* coding sequence itself.

In line with this, *DFR* knockout in the green leaf cultivar ‘Green Wave’ did not result in visually detectable phenotypic changes. This observation suggests that, under the conditions examined in this study, *DFR* knockout does not cause obvious morphological alterations in green leaf lettuce.

### 4.2 Metabolic flux redirection and flavonol accumulation

The inhibition of anthocyanin biosynthesis is presumed to redirect the metabolic flux of dihydroflavonols (e.g., DHK) toward FLS (flavonol synthase). DFR (dihydroflavonol 4-reductase) and FLS share common substrates such as dihydrokaempferol (DHK), dihydroquercetin (DHQ), and dihydromyricetin (DHM) and are known to compete for them (Choudhary and Pucker, 2024; Lei et al., 2023; Figure 1A); thus, this kind of metabolic rerouting is theoretically plausible. Indeed, marked increases in flavonols were observed in lines R1-25 and R1-62, supporting this interpretation. Although the flavonol content increased, the slight decrease in the total phenolic content may reflect the high contribution of anthocyanins to the Folin–Ciocalteu assay signal (Everette et al., 2010) or the limited compensatory flux toward other phenolic subclasses.

Similar phenotypic consequences of *DFR* mutations have been described in other species. In petunia, natural and induced mutations in *DFR* lead to the complete loss of anthocyanins, resulting in white-flowered cultivars but without a notable redirection of flux toward flavonols (Holton and Cornish, 1995; Meyer et al., 1987). In contrast, *Arabidopsis DFR* (also known as *TT3*) mutants not only lose anthocyanins but also exhibit increased accumulation of upstream flavonols, accompanied by altered stress responses (Peer and Murphy, 2007; Shirley et al., 1992). In tomato, targeted suppression of *DFR* reduces fruit pigmentation but often results in partial metabolic compensation via other branches of the flavonoid pathway (Bovy et al., 2002; Luo et al., 2008). These interspecies comparisons suggest that the metabolic consequences of *DFR* loss can differ depending on the inherent regulatory networks and tissue-specific roles of flavonoids. Our observation that red lettuce *DFR*-KO lines displayed a marked increase in flavonols is therefore consistent with the *Arabidopsis* model but also highlights the potential to exploit this metabolic rerouting in leafy vegetables cultivated under controlled PFAL conditions.

Although trace-level anthocyanin signals were detected in *DFR*-knockout lines (Figure 5C), these values were close to the detection limit of the assay and did not correspond to visible pigmentation. Therefore, they do not represent functional anthocyanin accumulation. The absence of visible coloration and the stable genotypes observed in T_2_ lines indicate that *DFR* function was effectively disrupted.

It should also be noted that flavonoid accumulation can vary even among lines carrying comparable DFR knockout alleles (e.g., R1-11 and R1-25 in Figure 5). In particular, the R1-11 line exhibited a distinct pattern in total flavonoid content compared with the other edited lines. At present, we cannot determine the decisive cause(s) of this line-specific outcome. Plausible, non-mutually exclusive explanations include (i) line-specific effects introduced during tissue culture (e.g., somaclonal/background variation), (ii) epigenetic differences affecting pathway responsiveness, and/or (iii) subtle microenvironmental heterogeneity within the PFAL (e.g., position-dependent gradients in light intensity/spectrum and temperature) that may influence flavonoid partitioning. Therefore, we avoid over-interpreting the R1-11 pattern mechanistically. Nevertheless, such variability may provide a useful clue for future studies aimed at identifying the determinants that may modulate anthocyanin/flavonoid allocation in *DFR*-knockout lettuce and establishing more efficient strategies for controlling pigmentation-related traits under PFAL conditions.

### 4.3 Minimal impact on growth and potential for PFAL breeding

Alterations in pigment metabolism can potentially affect plant growth or photosynthesis; however, the morphology and dry weight of the *DFR*-knockout lines were comparable to those of the wild type, even in the T_2_ generation. This suggests minimal adverse effects on growth. Although no detectable growth penalty was observed, a slight tendency toward reduced growth was observed in the *DFR*-KO lines. These findings suggest that the importance of anthocyanin accumulation in shoot apices for protection against light-induced stress cannot be completely excluded.

Interestingly, previous studies have reported that *ANS* (anthocyanidin synthase) knockout mutations cause growth retardation in lettuce (Gurdon et al., 2021). The milder impact observed in our *DFR*-knockout lines might be due to the earlier blockade of the anthocyanin pathway, resulting in less disruption of overall flavonoid metabolism.

In *Arabidopsis*, *TT3* (*DFR*) mutants lack both anthocyanins and proanthocyanidins (seed coat tannins), resulting in pale, transparent testa phenotypes (Peer and Murphy, 2007; Shirley et al., 1992). The absence of these compounds reduces the oxidative protective capacity of the seed coat, thereby affecting seed longevity and the uniformity of germination (Pourcel et al., 2005). Moreover, although metabolic flux in anthocyanin/proanthocyanidin*-*deficient mutants is redirected toward flavonol production, this compensation remains insufficient for effective ROS scavenging (Peer and Murphy, 2007). As a result, these mutants exhibit reduced tolerance to photooxidative stress under high light and UV conditions (Nakabayashi et al., 2014). Collectively, these findings demonstrate that *DFR* plays multifaceted roles in stress responses and is directly linked to plant adaptive capacity, a point that should not be overlooked.

Although anthocyanins are involved in stress responses, their necessity may be lower in closed systems such as PFALs. Thus, these lines may be useful as breeding materials for PFAL-oriented applications.

### 4.4 Low genome editing efficiency in red lettuce and its potential causes

Despite using the same vector (pDeCas9-Kan, UBQ (pUbi in Supplementary Figure S2) promoter), genome editing was less efficient in red leaf lettuce (‘Red Fire’) than in green leaf lettuce (‘Green Wave’). This may reflect differences in Cas9 expression during callus culture or oxidative stress and transcriptional silencing caused by the high polyphenol environment. Oxidative browning caused by the accumulation and oxidation of phenolic compounds is a major impediment to *in vitro* culture, leading to growth inhibition and reduced regeneration capacity (Amente and Chimdessa, 2021; Jones and Saxena, 2013; Liu et al., 2024; Mahmoud et al., 2024). These phenomena are attributed to oxidative stress and phenolic compound metabolism interfering with cell viability and regeneration, providing a conceptual basis for interpreting the reduced editing efficiency observed in the high-polyphenol environment of red leaf lettuce.

Recent studies suggest that the AtUBQ10 and CaMV 35S promoters are less stable in lettuce, whereas endogenous promoters such as LsEF1α and LsACT2 may offer improved expression (Pan et al., 2022). This represents an important consideration for future vector design.

### 4.5 Regulatory and breeding implications: achieving non-GMO-type metabolic control

The introduced mutations were small insertions or deletions without foreign genes, corresponding to SDN-1-type editing. In Japan and some other countries, such mutations may be exempt from GMO regulations, lowering the barrier to commercializing GE crops (Ezura, 2022).

In addition, the clearly visible phenotype aids in not only selection efficiency but also explaining the edits to consumers and regulatory bodies. The clear effects of targeting a single gene, *DFR*, on pigmentation, composition, and growth highlight the practical value of rationally designed breeding.

### 4.6 Future perspectives

This study demonstrated metabolic reprogramming via *DFR* knockout; however, further investigation is needed. First, a transcript analysis of *FLS*, *ANS*, and *CHS* is needed to be performed to clarify whether the metabolic shift was driven by transcriptional regulation or enzyme-level changes. Second, physiological validation through antioxidant assays (e.g., DPPH) will be useful for evaluating changes in nutritional function.

Finally, combining this genetic approach with known environmental cues (e.g., light quality, UV, and temperature) may enable compensatory activation of flavonoid biosynthesis under controlled environmental conditions (Bian et al., 2015; Gould, 2004; Zoratti et al., 2014). Notably, red leaf lettuce cultivars have been reported to exhibit dramatic fluctuations in leaf anthocyanin content in response to environmental factors such as light intensity (Baek et al., 2013; Ouzounis et al., 2015; Palsha et al., 2024). Palsha et al. (2024) demonstrated that when six lettuce cultivars were grown under different photosynthetic photon flux densities (PPFDs) in greenhouse conditions, red leaf lettuce types showed pronounced changes in both anthocyanin accumulation and light-use efficiency depending on light intensity (Palsha et al., 2024). Therefore, in *DFR*-KO lettuce, strategically providing environmental stimuli that would normally drive anthocyanin biosynthesis may further increase the flavonol accumulation observed in this study. Such precise light control can be relatively easily achieved in PFAL systems, and a synergistic effect between *DFR*-KO lines and PFAL cultivation can thus be anticipated. Such integrative strategies, i.e., “genetic modification × environmental control”, may open new directions for functional crop production in a PFAL and other high-precision agricultural systems. These findings highlight the potential of genome editing to tailor flavonoid metabolism in leafy vegetables, aligning with the nutritional and aesthetic demands of PFAL-based production systems.

In the present study, no clear growth-promoting effect of *DFR* knockout was detected under the experimental conditions used. This may reflect the strong influence of environmental factors, such as light intensity and quality, on the balance between photoprotection and photosynthetic efficiency. Therefore, the potential contribution of anthocyanin modulation to growth performance remains an important topic for future investigation under controlled and well-defined environmental conditions.

## 5 Conclusion

Targeted editing of *DFR* resulted in a clear visual phenotype alteration and a shift in assay-based flavonoid/phenolic profiles in red leaf lettuce, with no obvious growth penalties under the tested PFAL condition. These results demonstrate that CRISPR/Cas9-mediated modification of a key flavonoid-pathway gene can reproducibly alter leaf pigmentation and associated metabolite readouts, providing a useful platform for further cultivar design and evaluation in controlled environments such as PFALs.

## Data Availability

The datasets presented in this study can be found in online repositories. The names of the repository/repositories and accession number(s) can be found in the article/Supplementary Material.
