# Supplementary material for: CRISPR/Cas9-mediated knockout of DFR alters pigmentation and shifts flavonoid accumulation in red leaf lettuce without detectable growth penalties
Source: Front Genome Ed. 2026 Mar 4;8:1755922. doi: 10.3389/fgeed.2026.1755922 (PMC12996048; doi:10.3389/fgeed.2026.1755922)
Supplement: Supplementary file 1 [file DataSheet1.PDF]

Supplementary Material

1 Supplementary Tabel 1.

PrimerID	Sequences	Amplicon size (bp)	Template	Gene name	Memo
ANg5-F	ATTGTGGTGTTTACATCCTCTGCG	-	Lsat_1_v5_gn_2_77261_guide 2	DFR	Guide RNA for DFR gene: guide2 on CRISPR-P result
ANg5-R	AAACCGCAGAGGATGTAAACACCA	-	Lsat_1_v5_gn_2_77261_guide 2	DFR	Guide RNA for DFR gene: guide2 on CRISPR-P result
ANg6-F	ATTGTTATGAGACTTCTTGAACGT	-	Lsat_1_v5_gn_2_77261_guide 4	DFR	Guide RNA for DFR gene: guide4 on CRISPR-P result
ANg6-R	AAACACGTTCAAGAAGTCTCATAA	-	Lsat_1_v5_gn_2_77261_guide 4	DFR	Guide RNA for DFR gene: guide4 on CRISPR-P result
Cas9 F1	AGTACGTGACCGAGGGAATG	207	T-DNA (pDeCas9-Kan)	Cas9	T-DNA detection
Cas9 R1	GATCGTGGTAGGTTCCGAGA	207	T-DNA (pDeCas9-Kan)	Cas9	T-DNA detection
ON3-F	GAAAGAGGATTCTCCGACCACC	373	Lsat_1_v5_gn_2_77261	DFR	Sequencing primer for guide4 read
ON3-Rr	GCGTAGCCACATGAAAGACTC	373	Lsat_1_v5_gn_2_77261	DFR	Sequencing primer for guide4 read
ON2-Fr	GCCAACAATAGAAGGTGTATTAAGC	194	Lsat_1_v5_gn_2_77261	DFR	Sequencing primer for guide2 read
ON2-R	TGAAACGAACCCATGCAGTC	194	Lsat_1_v5_gn_2_77261	DFR	Sequencing primer for guide2 read

Supplementary Table 1. List of oligo sequences which are used in this paper.

2 Supplementary Figures. 1

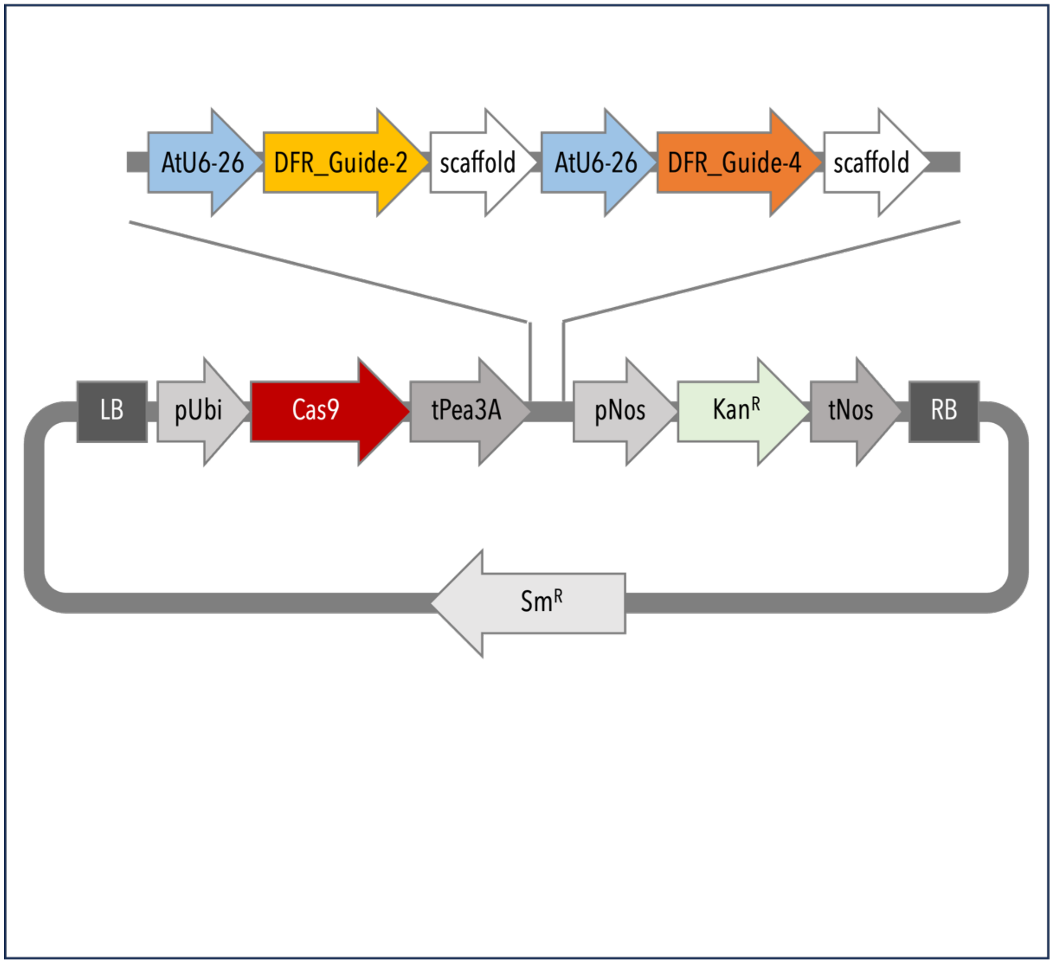

Supplementary Figure 2. Binary vector structure for DFR knockout in lettuce.
